# 500 metagenome-assembled microbial genomes from 30 subtropical estuaries in South China

**DOI:** 10.1038/s41597-022-01433-z

**Published:** 2022-06-16

**Authors:** Lei Zhou, Shihui Huang, Jiayi Gong, Peng Xu, Xiande Huang

**Affiliations:** 1 Joint Laboratory of Guangdong Province and Hong Kong Region on Marine Bioresource Conservation and Exploitation, College of Marine Sciences, South China Agricultural University, Guangzhou, China; 2 Guangxi Key Laboratory of Beibu Gulf Marine Biodiversity Conservation, College of Marine Sciences, Beibu Gulf University, Qinzhou, 535011 China

**Keywords:** Water microbiology, Microbial ecology

## Abstract

As a unique geographical transition zone, the estuary is considered as a model environment to decipher the diversity, functions and ecological processes of microbial communities, which play important roles in the global biogeochemical cycle. Here we used surface water metagenomic sequencing datasets to construct metagenome-assembled genomes (MAGs) from 30 subtropical estuaries at a large scale along South China. In total, 500 dereplicated MAGs with completeness ≥ 50% and contamination ≤ 10% were obtained, among which more than one-thirds (n = 207 MAGs) have a completeness ≥ 70%. These MAGs are dominated by taxa assigned to the phylum Proteobacteria (n = 182 MAGs), Bacteroidota (n = 110) and Actinobacteriota (n = 104). These draft genomes can be used to study the diversity, phylogenetic history and metabolic potential of microbiota in the estuary, which should help improve our understanding of the structure and function of these microorganisms and how they evolved and adapted to extreme conditions in the estuarine ecosystem.

## Abstract (summary)

**Table:** 

Measurement(s)	Metagenome-assembled genomes
Technology Type(s)	Metagenomics
Sample Characteristic - Organism	Bacteria • Archaea
Sample Characteristic - Environment	estuarine water
Sample Characteristic - Location	South China Sea coastal waters of the mainland of China

## Background & Summary

The estuary is the intersection of fresh water, land and sea water, where fresh water and sea water with different properties are mixed, and a large number of nutrients and terrestrial microbes are input and accumulated. The complex condition leads to diverse biocoenosis in the estuarine environment. Microorganisms, such as Bacteria and Archaea, are widely distributed, abundant, and play key roles in biogeochemical cycle of carbon, nitrogen, sulfur and phosphorus as well as microbial food web in estuarine ecosystems^1–3^. As one of the most productive ecosystems in the world^4^, the strong natural and anthropogenic gradients in estuaries make them ideal niches to study microbial community structure and its associated functions.

The recent development of high-throughput sequencing technology such as 16 S rRNA gene and metagenome sequencing can identify large amounts of unknown taxa, analyze the characteristics of uncultured microorganisms, and thus has promoted studies of microbial diversity, community assembly, adaptation, evolution and function^2,3^. The research of microbial community structure in various estuaries such as Chesapeake Bay^5^, Delaware estuary^6^, Columbia estuary^7^, and estuaries of Sundarbans (i.e., Mooriganga, Thakuran, Matla, and Harinbhanga)^8^ has been carried out. Microbiological studies have also been conducted in several major estuaries in China, such as Yellow River, Yangtze River, Qiantang River and Pearl River^1,9–12^. These studies provided insights into spatial-temporal variations of microbial communities and their responses to environmental changes in estuarine ecosystems. However, despite the increasing knowledge of biodiversity process in the estuarine ecosystem, our understanding of the distributions and ecological preferences and functions of estuarine microbiome across broad spatial scales remains surprisingly limited.

**Fig. 1:**
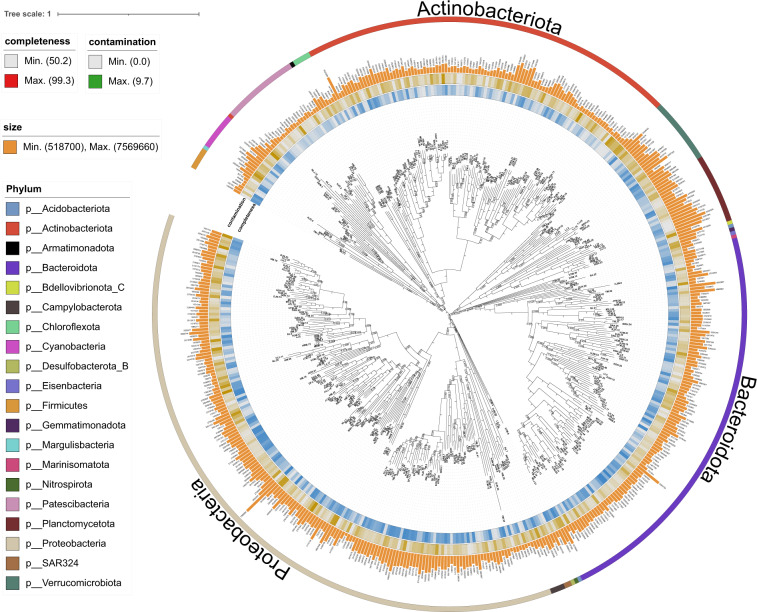
Phylogenetic tree of the MAGs constructed by maximum likelihood method using a concatenated alignment of 120 conserved bacterial markers. Concentric rings moving outward from the tree show the completeness, and contamination and inferred phylum. The bar plot shows the size of the MAGs.

Here we present 500 metagenome-assembled genomes (MAGs) reconstructed from 90 surface water metagenomic samples in 30 subtropical estuaries which span the estuary of 30 major rivers in Guangdong and Guangxi, South China, a range of ~1300 km. All of these MAGs were estimated to be > 50% complete with < 10% contamination. Among them, 41.40% (207) have a completeness > 70% and 13.20% (66) have a completeness > 90%, while 75.80% (379) have low ( < 5%) contamination and 4.00% (20) have no contamination. Together, high-quality MAGs (Completion > 90% and Contamination < 5%) account for 12.2% (61) and medium-quality MAGs (Completion ≥ 50% and Contamination < 10%) account for 87.8% (439). The draft genomes were classified into 491 bacteria and 9 archaea. A vast majority of them belong to the phyla Proteobacteria (36.4%), Bacteroidota (22%), and Actinobacteria (20.8%) (Table 1; Fig. 1). However, only 62 (12.4%) could be classified to current known taxa at species level with 438 (87.6%) representing currently uncultured species. For fully utilizing the genome data, statistics of quality control on metagenomic raw reads is provided in Supplementary Table S1. Assembly information is provided in Supplementary Table S2. Predicted taxon for each MAG, as well as bin statistics (e.g., completeness, contamination, size and N50), are provided in Supplementary Table S3. MAGs abundance in each estuary is provided in Supplementary Table S4 and associated environmental variables is given in Supplementary Table S5.

**Table 1:** Relative proportion of phyla in MAGs reconstructed from the subtropical estuaries, South China.

Domain	Phylum	Count	Propotion (%)
d__Archaea	p__Thermoplasmatota	6	1.2
	p__Thermoproteota	3	0.6
d__Bacteria	p__Proteobacteria	182	36.4
	p__Bacteroidota	110	22
	p__Actinobacteriota	104	20.8
	p__Patescibacteria	21	4.2
	p__Planctomycetota	19	3.8
	p__Verrucomicrobiota	18	3.6
	p__Cyanobacteria	10	2
	p__Firmicutes	6	1.2
	p__Chloroflexota	5	1
	p__Campylobacterota	4	0.8
	p__SAR324	2	0.4
	p__Acidobacteriota	2	0.4
	p__Margulisbacteria	1	0.2
	p__Armatimonadota	1	0.2
	p__Bdellovibrionota_C	1	0.2
	p__Marinisomatota	1	0.2
	p__Gemmatimonadota	1	0.2
	p__Nitrospirota	1	0.2
	p__Desulfobacterota_B	1	0.2
	p__Eisenbacteria	1	0.2

To the best of our knowledge, this is the largest number of microbial genomes from the largest number of estuaries to be reported in a single study, which should help facilitate future studies in understanding the structure and function of these microorganisms and how they evolved and adapted to the extreme conditions of the estuarine ecosystems.

## Methods

### Sample sites and sample collection

**Fig. 2:**
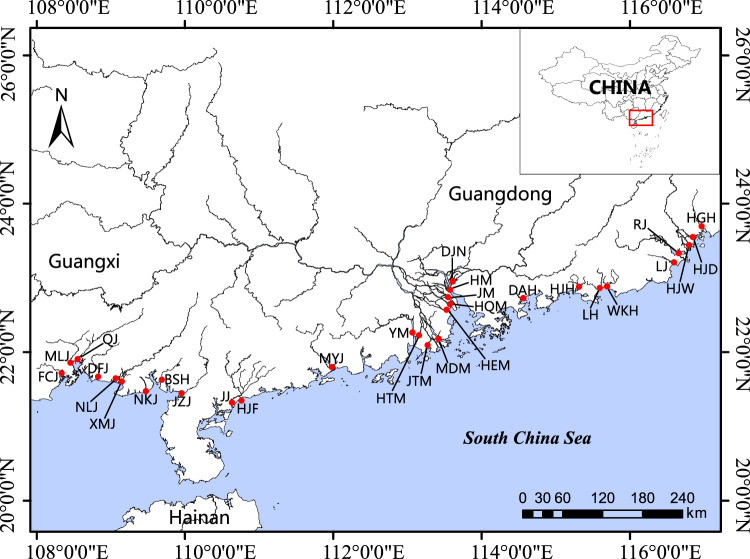
Map of the sampling estuaries. HGH, Huanggang river estuary; HJD, Hanjiangdong river estuary; HJW, Hanjiangwaisha river estuary; RJ, Rongjiang River river estuary; LJ, Lianjiang river estuary; WKH, Wukanhe river estuary; LH, Luohe river estuary; HJH, Huangjianghe river estuary; DAH, Danaohe river estuary; DJN, Dongjiangnan river estuary, HM, Humen mouth; JM, JiaoMen mouth; HQM, Hongqimen mouth; HEM, Hengmen mouth; MDM, Modaomen mouth; JTM, Jitimen mouth; HTM, Hutiaomen mouth; YM, Yamen mouth; MYJ, Moyangjiang river estuary; HJF, Huangjiangfengonghe river estuary; JJ, Jianjiang river estuary; JZJ, Jiuzhoujiang river estuary; BSH, Baishahe river estuary; NKJ, Nankangjiang river estuary; NLJ, Nanliujiang river estuary; DFJ, Dafengjiang river estuary; QJ, Qinjiang river estuary; MLJ, Maolingjiang river estuary; FCJ, Fangchengjiang river estuary; XMJ, Ximenjiang river estuary.

A total of 90 surface water samples were collected in December 2018 from 30 sites that spanned the estuary of 30 main rivers in South China, a range of ~1300 km (Fig. 2). At each estuary, triplicate samples were collected, approximately 30–50 m apart. 500 mL water was filtered for the metagenome sequencing through 0.22-μm pore polycarbonate membranes (Millipore Corporation, Billerica, MA, USA), as most prokaryotes are larger than that size. The filtration was performed within 4~8 h and the filter membranes were quick-frozen in liquid nitrogen and then stored at −80 °C until DNA extraction.

### DNA extraction, metagenomic sequencing and assembly

Total microbial DNA was extracted using a FastDNA Spin Kit for Soil (MP Biomedicals, CA, USA) following the manufacturer’s instructions. The quality and concentration of extracted DNA were evaluated by agarose gel electrophoresis (1%) and Qubit® dsDNA Assay Kit in Qubit® 2.0 Flurometer (Life Technologies, CA, USA). All extracted DNA was stored at −20 °C for further applications.

A total amount of 1 μg DNA per sample was used as input material for the sequencing preparations. Sequencing libraries were generated using NEBNext® Ultra™ DNA Library Prep Kit for Illumina (NEB, USA) following manufacturer’s recommendations and index codes were added to attribute the sequences to each sample. Briefly, the DNA sample was fragmented by sonication to a size of 350 bp, then DNA fragments were end-polished, A-tailed, and ligated with the full-length adaptor for Illumina sequencing with further PCR amplification (2 circles). At last, PCR products were purified (AMPure XP system) and libraries were analyzed for size distribution by Agilent2100 Bioanalyzer. After cluster generation, the library preparations were sequenced (Paired-end 2 × 150 bp) on an Illumina NovaSeq. 6000 platform in Microeco, Shenzhen, China. After sequencing, the raw reads were filtered using kneadData v0.7.4. (https://bitbucket.org/biobakery/kneaddata/wiki/Home) with options (–trimmomatic-options “ILLUMINACLIP: TruSeq. 2-PE.fa:2:40:15 SLIDINGWINDOW:4:20 MINLEN:50”–bowtie2-options “–very-sensitive- dovetail -db Homo_sapiens”). About 6 Gb (giga base pairs) of clean metagenomic data was generated for each sample, resulting in a total of ~580 Gbp data. Trimmed metagenomic reads were co-assembled for samples from the same estuary using MEGAHIT v1.2.9 with the default settings^13^. The quality of the metagenomic assemblies assessed with tools like metaQUAST v 5.0.2^14^.

### Genome binning and refinement

Genome binning and refinement were all conducted in metaWRAP 1.3^15^. In details, contigs were clustered into metagenomic bins using metaWRAP binning module (–maxbin2–concoct–metabat2 options). The resulting bins were then refined with metaWRAP’s bin_refinement module (-c 50 -x 10 options). To increase the completion of the bins, and reduce contamination, metaWRAP reassemble_bins module(-c 50 -x 10 options) was used by extracting the reads belonging to each bin, and reassembling the bins with SPAdes v3.10.1 with the–carefull setting^16^. These decontaminated bins were then dereplicated using dRep v2.6.2^17^ with parameters: -sa 0.95 -nc 0.30 -comp 50 -con 10. The bins were then quantified with the Quant_bins module (default parameters)^18^. First, Salmon v0.13.1^19^ (quasi-mapping-based mode–libType IU–meta options) was used to produce abundance values (TPM) for each contig. Then, the overall abundance of the bin in each sample was calculated by taking the length-weighted average of the contig abundances.

### Taxonomic classification and genome tree construction

The taxonomy of the 500 MAGs (bins) were classified using GTDB-Tk v1.3.0^20^ with the GTDB r202^21^. Phylogenetic relationships among the 491 bacterial MAGs or nine archaeal MAGs were inferred by constructing a maximum-likelihood tree using 120 bacterial and 122 archaeal marker genes identified in GTDB-Tk. In detail, bacterial and archaeal reference trees are inferred from the filtered 120 and 122 phylogenetically informative markers, respectively. The bacterial reference tree is inferred with FastTree v2.1.10^22^. under the WAG model. The archaeal reference tree is inferred with IQ-Tree v1.6.9^23^ under the PMSF model, a rapid approximation of the C10 mixture model (LG + C10 + F + G), using FastTree v2.1.10 to infer an initial guide tree. Both trees contain non-parametric bootstrap support values. The tree was viewed and annotated using Itol^24^ (https://itol.embl.de).

## Data Records

The raw sequence data are available on the NCBI Sequence Read Archive (PRJNA730330)^25^. 500 MAGs, the genome trees are available in figshare^26^. They have been appropriately specified in the text where required.

## Technical Validation

To validate the completeness and contamination of the genomes, we accessed the number of marker genes present in all MAGs using CheckM v1.1.3^27^ (checkm lineage_wf–tab_table -g -x faa -e 1e-10 -l 0.7). It provides robust estimates of genome completeness and contamination by using collocated sets of genes that are ubiquitous and single-copy within a phylogenetic lineage. Completeness and contamination scores are estimated by detecting the presence and number of single-copy marker genes in the draft genome. An uncontaminated and complete MAG will have all of these marker genes present just once in the genome. This final catalog comprises of only those genomes that met specific quality thresholds (i.e., completeness ≥ 50% and contamination < 10%) as described in the manuscript. Additionally, to improve the quality (i.e., increasing completion and reducing contamination), the bins were reassembled in metaWRAP.

## Supplementary information

Additional information Table S1, Table S2, Table S3, Table S4, Table S5

## Data Availability

Custom scripts were not used to generate or process this dataset. Software versions and non-default parameters used have been appropriately specified where required.
